# Collecting Maternal Health Information From HIV-Positive Pregnant Women Using Mobile Phone-Assisted Face-to-Face Interviews in Southern Africa

**DOI:** 10.2196/jmir.2207

**Published:** 2013-06-10

**Authors:** Alastair van Heerden, Shane Norris, Stephen Tollman, Linda Richter, Mary Jane Rotheram-Borus

**Affiliations:** ^1^Human Sciences Research CouncilPietermaritzburgSouth Africa; ^2^School of Public HealthFaculty of Health SciencesUniversity of the WitwatersrandJohannesburgSouth Africa; ^3^MRC/WITS Developmental Pathways for Health Research UnitDepartment of Paediatrics, School of Clinical MedicineUniversity of the WitwatersrandJohannesburgSouth Africa; ^4^MRC/Wits Rural Public Health and Health Transitions Research Unit (Agincourt)School of Public Health, Faculty of Health SciencesUniversity of the WitwatersrandJohannesburgSouth Africa; ^5^Centre for Global Health ResearchUmeå UniversityUmeåSweden; ^6^INDEPTH NetworkAccraGhana; ^7^Global Center for Children and FamiliesUniversity of California at Los AngelesLos Angeles, CAUnited States

**Keywords:** mobile phones, human immunodeficiency virus, mobile health

## Abstract

**Background:**

Most of the world’s women living with human immunodeficiency virus (HIV) reside in sub-Saharan Africa. Although efforts to reduce mother-to-child transmission are underway, obtaining complete and accurate data from rural clinical sites to track progress presents a major challenge.

**Objective:**

To describe the acceptability and feasibility of mobile phones as a tool for clinic-based face-to-face data collection with pregnant women living with HIV in South Africa.

**Methods:**

As part of a larger clinic-based trial, 16 interviewers were trained to conduct mobile phone–assisted personal interviews (MPAPI). These interviewers (participant group 1) completed the same short questionnaire based on items from the Technology Acceptance Model at 3 different time points. Questions were asked before training, after training, and 3 months after deployment to clinic facilities. In addition, before the start of the primary intervention trial in which this substudy was undertaken, 12 mothers living with HIV (MLH) took part in a focus group discussion exploring the acceptability of MPAPI (participant group 2). Finally, a sample of MLH (n=512) enrolled in the primary trial were asked to assess their experience of being interviewed by MPAPI (participant group 3).

**Results:**

Acceptability of the method was found to be high among the 16 interviewers in group 1. Perceived usefulness was reported to be slightly higher than perceived ease of use across the 3 time points. After 3 months of field use, interviewer perceptions of both perceived ease of use and perceived usefulness were found to be higher than before training. The feasibility of conducting MPAPI interviews in this setting was found to be high. Network coverage was available in all clinics and hardware, software, cost, and secure transmission to the data center presented no significant challenges over the 21-month period. For the 12 MHL participants in group 2, anxiety about the multimedia capabilities of the phone was evident. Their concern centered on the possibility that their privacy may be invaded by interviewers using the mobile phone camera to photograph them. For participants in group 3, having the interviewer sit beside vs across from the interviewee during the MPAPI interview was received positively by 94.7% of MHL. Privacy (6.3%) and confidentiality (5.3%) concerns were low for group 3 MHL.

**Conclusions:**

Mobile phones were found both to be acceptable and feasible in the collection of maternal and child health data from women living with HIV in South Africa.

**Trial Registration:**

Clinicaltrials.gov NCT00972699; http://clinicaltrials.gov/ct2/show/NCT00972699 (Archived by WebCite at http://clinicaltrials.gov/ct2/show/NCT00972699)

## Introduction

One-half of people living with human immunodeficiency virus (HIV) globally are women and 76% of all HIV-positive women live in sub-Saharan Africa [1]. Sub-Saharan Africa accounts for almost half of the world’s maternal, newborn, and child deaths with 4.7 million children and 276,000 women dying annually [2,3]. Mothers living with HIV (MLH) are particularly vulnerable and at risk of adverse maternal outcomes with at least 20% of maternal deaths being HIV-related [1]. South Africa makes up a large proportion of this disease burden with 3.2 million women living with HIV [1], of whom 200,000 annually are pregnant [4]. The Province of KwaZulu-Natal has the highest HIV prevalence in South Africa [5]. The national prevalence is approximately 11% [5], whereas 40% to 60% of pregnant women in rural KwaZulu-Natal are HIV-positive [6,7].

In response to this crisis, South Africa has implemented the Prevention of Maternal-to-Child Transmission (PMTCT) package as recommended by the World Health Organization (WHO). The PMTCT program requires newly pregnant women to complete a series of sequential steps, also known as the PMTCT cascade [8], that are aimed at first diagnosing and then treating HIV infection [9]. Under ideal circumstances, in which no barriers exist to the completion of all tasks, PMTCT has been shown to be highly effective at reducing HIV transmission to less than 2% at childbirth [10]. Under the less than ideal circumstances faced by many MLH in low- and middle-income countries, transmission can occur in approximately 1 in 4 deliveries [11]. Some of the challenges that make adherence to each of the PMTCT tasks difficult are being unable to afford transportation to the clinic, fear of stigmatization, increased household conflict, and lack of partner support [8]. To better understand these barriers and their impact on the loss of women through the PMTCT cascade requires accurate, timely, and detailed information. Yet gathering data that tracks women’s progress through PMTCT to determine how well the system is performing is beset by its own challenges.

One of the major challenges faced in the collection of high-quality data is human resource constraints. Trained and qualified health staff are in short supply in many resource-constrained settings [12]. Task-shifting strategies used to address these shortages, have led to a perceived increased burden for data collection and collation [13]. Minimal support, delayed feedback, little understanding of the usefulness of the data, and no interpretation of raw scores mean that the completion and submission of multiple paper-based registers for statistical purposes is often regarded as a low priority for busy and overburdened staff [13]. Therefore, it is unsurprising that register data has been shown to be fragmented, error prone, incomplete, and inaccessible [14,15].

The usefulness of this register data is further compromised by the fact that it is unlinked, ie, it is not possible to link individual clinic visits to the same person. This means that although aggregate data may be available, it is difficult to track the path of individuals through the PMTCT cascade. This makes it difficult to identify and understand the bottlenecks in the cascade. These constraints and challenges raise the question whether there is not a more efficient way to support staff and perform monitoring and evaluation of the PMTCT program in geographically remote primary health care facilities [16].

There is currently a groundswell of interest in the use of mobile phones and information communication technologies in the support of health [17,18]. The growing body of mHealth literature provides examples of the use of mobile phones as data collection tools in low- and middle-income countries. Although evidence is mixed, the use of mobile phones as data collection tools has been found to increase data quality, speed up the turnaround time from collection to analysis, and improve interfacility communications [19-22]. Poor follow-up rates could potentially be improved with mobile phones by providing links to participants that are cheaper and more immediate than travel to facilities. These advantages over traditional paper-based clinic registers suggest mobile phones are a potential tool with which to address some of the challenges currently experienced in collecting health information through the PMTCT cascade. The aim of this paper is to describe the feasibility and acceptability of using a mobile phone survey application to collect data from pregnant women living with HIV enrolled into the PMTCT program in KwaZulu-Natal, South Africa.

## Method

### Study Design

This study was nested within a larger clinic-based randomized cluster trial known as Project Masihambisane (“let us walk together”; Clinicaltrials.gov NCT00972699) [23]. The primary study aimed to improve mental and physical health outcomes of HIV-positive mothers and their babies by supplementing the PMTCT with paraprofessional peer mentors. Using a mixed-methods design, qualitative data from a single, small focus group were supplemented with 2 quantitative questionnaires collected using a mobile phone survey application. Paradata, or data on the data collection process, were gathered in order to examine the feasibility of mobile phone-assisted personal interviewing (MPAPI).

### Mobile Phone-Assisted Personal Interviewing

**Figure 1:**
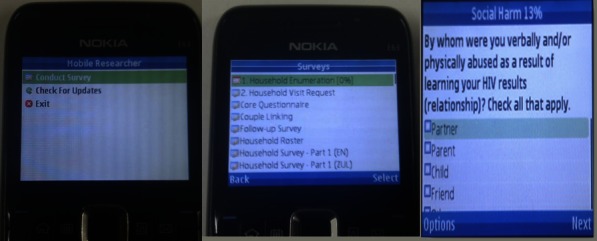
Example of the Mobenzi Researcher application running on a Nokia handset.

The MPAPI survey platform was supplied by Mobenzi Researcher [24], a commercial vendor based in South Africa. The solution offered by Mobenzi includes both a mobile application and a Web portal. The Java Platform Micro Edition (Java ME) application runs on all handsets compliant with mobile information device profile (MIDP) 2.0. It provides full survey functionality, including the ability to create various question types, mark fields as mandatory, and intelligently manage survey branching (Figure 1). The software is now also available for Android handsets. The Java ME application was installed on Nokia E61 handsets. These mobile phones run on the Symbian S60 operating system, have a 2.9-inch thin film transistor screen, 64 megabyte random-access memory (RAM), Bluetooth, Wi-Fi, a QWERTY keyboard, and a 1500 milliamp hour battery.

**Figure 2:**
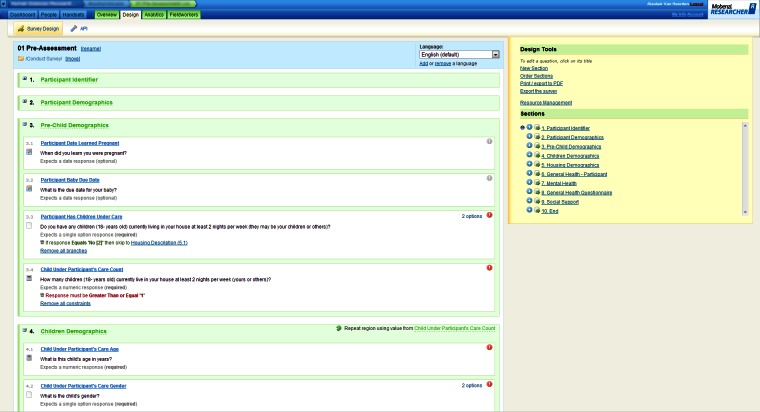
Example of Mobenzi Researcher Web portal: survey design.

**Figure 3:**
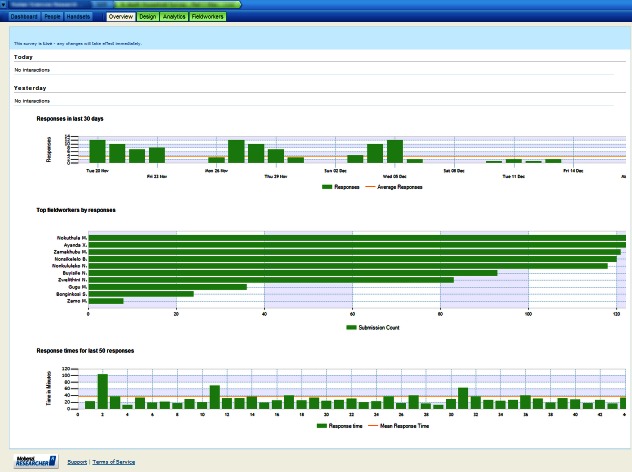
Example of Mobenzi Researcher Web portal: interviewer management.

**Figure 4:**
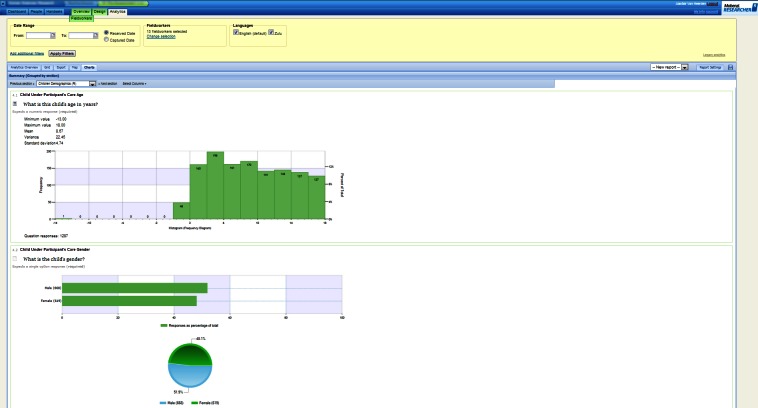
Example of Mobenzi Researcher Web portal: charting and analytics.

**Figure 5:**
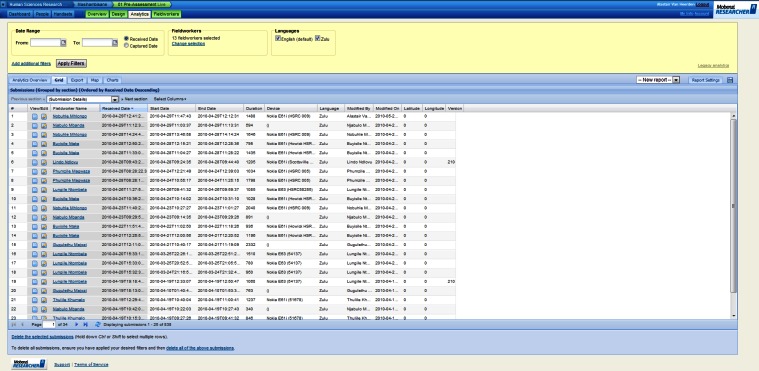
Example of Mobenzi Researcher Web portal: data overview and export.

Once installed, the software was able to communicate, using either Wi-Fi or a cellular data link, with the Mobenzi server. The server provided, for download, the surveys designed using the Web portal (Figure 2). The server also received, stored, and aggregated the surveys completed on the handset (Figures 3-5) for download as a comma-separated file. Mobenzi offered programmatic access to both surveys and data through an application programming interface (API).

**Figure 6:**
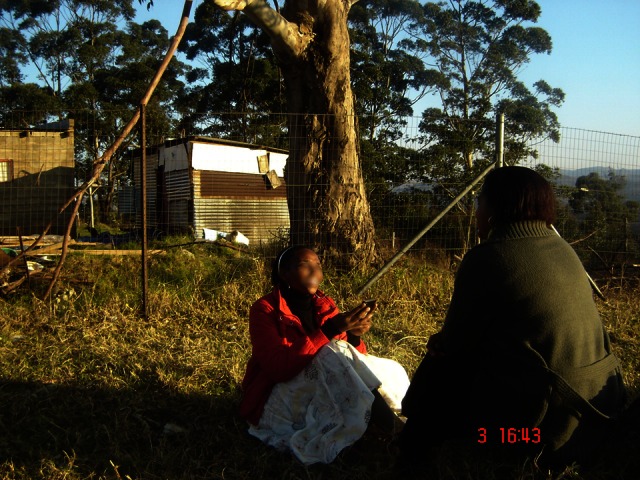
Example of Mobenzi Researcher in field use.

If no data connection was available at the time of survey completion, the response was saved on the handset until a connection was re-established. The MTN mobile network was used to upload survey responses from the handset to server. Figure 6 depicts a typical example of a fieldworker conducting a mobile phone–assisted personal interview outside a primary health care facility.

### Participants and Procedures

Three groups of participants took part in this study. The first group of participants (participant group 1) included the 16 interviewers recruited and trained on MPAPI for the primary study. Although these participants were all familiar with pen-and-paper questionnaires, none had ever used MPAPI. Before receiving any training on MPAPI, the group were asked to complete a short questionnaire with items from the Technology Acceptance Model proposed by Davis [25]. The scale contains 12 items, 6 relating to the perceived usefulness of mobile phones for research purposes and 6 related to the perceived ease of their use (Table 1). Each item was rated on a 5-point Likert-type scale ranging from extremely likely to extremely unlikely. Following 3 days of training, a posttraining assessment was conducted using the same questionnaire. After completing training, the interviewers were dispatched to 8 primary health care clinics in a rural district typical of those found in the region where the interviewers were originally interviewing pregnant women living with HIV for the primary study. After 3 months, the questionnaire was administered for a third time to gauge if use of the phone in the field had altered perceptions and attitudes toward the tool.

The second group (participant group 2), recruited for participation in the focus group, consisted of 12 pregnant women with HIV living in this region of South Africa who were enrolled through community forums set up for the larger study. Before the focus group was held, each woman was interviewed by a research assistant using a mobile phone. The questions asked ranged from general health questions about the participants’ knowledge of HIV to more sensitive questions about recent sexual activity, condom use, and disclosure of HIV status to their partners. After completing the questionnaire, the group was brought together to participate in a focus group about their experience. Five questions were designed to facilitate the discussion about general mobile phone ownership and their reactions to the interview conducted by mobile phone. The focus group lasted for an hour with extensive notes being taken about responses.

The third group of participants (participant group 3) were pregnant women living with HIV recruited through the primary study. Participants were recruited in the Umgungundlovu Health District of KwaZulu-Natal, a province in South Africa with 10 million people, over half (57%) living in rural areas. The Umgungundlovu District includes 7 local authorities, has 48 fixed clinics, 4 community health centers, 9 tertiary hospitals, and an estimated population of some 995,000 persons according to a 2007 estimate [26]. From this district, 8 clinics were selected through a clinic audit conducted in December 2007. The selection criteria, applied to all potential clinics in the district during the audit, were presence of other research trials, clinic size, availability of antenatal and postnatal services, and uptake of antenatal and postnatal services at the clinic. The clinic audit resulted in the selection of 4 pairs of clinics for the study, matched on size (small vs large) and geography (rural vs urban). Women who met the eligibility criteria of being 18 years or older, less than 34 weeks pregnant, and planning to reside in the study area for the duration of their pregnancy were invited to participate in the primary study. If they accepted, a baseline health questionnaire was completed by an interviewer using a mobile phone survey application. Using a cross-sectional design and a continuous sampling strategy that started approximately halfway through the primary study, 512 of the 1204 women enrolled into the primary study were recruited to participate in this substudy. This cross-section of women completed a second questionnaire, again using MPAPI, in which they were asked to describe their views about the mobile phone survey.

### Statistical Analysis

A thematic analysis was performed on the qualitative data generated by focus group discussions. This data was supplemented with exploratory data analysis techniques, such as frequency analysis and chi-square (χ^2^) statistics, performed on the quantitative questionnaire data. Univariate analysis was used to analyze perceived ease of use and perceived usefulness scale data. Data was downloaded as comma-separated values from the online Mobile Research database. This comma-separated values file was then imported into SPSS 19 (IBM Corp, Armonk, NY, USA).

**Table 1:** Technology Acceptance Model scale.

Items per subscale		Question text
**Perceived usefulness**		
	1	Using cell phones to collect data in my job would enable me to accomplish tasks more quickly
	2	Using cell phones to collect data would improve my job performance
	3	Using cell phones to collect data in my job would increase my productivity
	4	Using cell phones to collect data would enhance my effectiveness on the job
	5	Using cell phones to collect data would make it easier to do my job
	6	I would find cell phones useful in my job
**Perceived ease of use**		
	1	Learning to operate a cell phone would be easy for me
	2	I would find it easy to get a cell phone to do what I want it to do
	3	My interaction with cell phones would be clear and understandable
	4	I would find cell phones flexible to interact with
	5	It would be easy for me to become skilful at using a cell phone
	6	I would find a cell phone easy to use

### Fieldwork Supervision and Ethical Approval

A team of 16 data collectors administered the questionnaire and 2 coordinators employed by the primary trial supervised all aspects of the study in the field. The coordinators’ role included managing informed consent, supporting field activities, and monitoring data quality through quality assurance checks and ongoing training and supervision of interviewers. Ethical approval for the study was obtained from the Committee for Research on Human Subjects (Medical) Witwatersrand Human Ethics Committee (M091035). A study information sheet was presented to all participants and the study was explained in detail before participants signed a form giving written consent to participate.

## Results

### Acceptability

#### Participant Group 1: Interviewers

Sixteen interviewers were recruited, trained, and dispatched to 8 health facilities as part of the primary trial. All participants were female with an average age of 27 years. Phone ownership was high with 15 of 16 owning their own mobile phone. All interviewers had completed their secondary education, with 14 having completed a 3-year tertiary degree. All had previous experience with paper-and-pen questionnaires, but none reported ever having performed a mobile phone–assisted interview.

**Figure 7:**
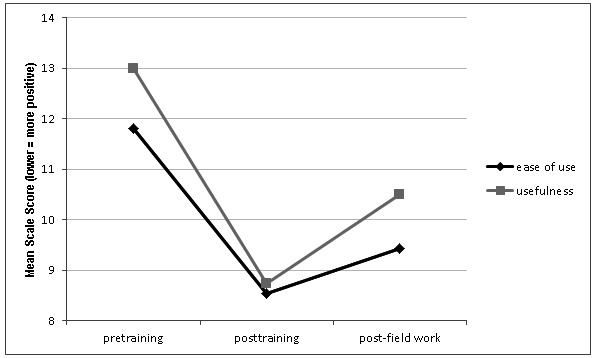
Estimated marginal means for the main effect of time.

A 2-way repeated measures ANOVA was performed on the 2-factor Technology Acceptance Model scale (ease of use and usefulness) across 3 time points (pretraining, posttraining, and postuse). There was a significant main effect for the time point at which the data was collected (*F*_1.189,16.649_=8.62, *P*=.007). However, there was no significant main effect between perceived ease of use and usefulness (*F*_1,14_=0.924, *P*=.35) nor any interaction effect between these factors and time (*F*_2,28_=0.621, *P*=.54). The findings are presented in Figure 7. A maximum score of 55 implies extreme dislike of the tool; therefore, interviewer attitudes toward ease of use and usefulness of mobile phones were already positive before training.

After 3 days of training, interviewers reported perceiving the mobile phones as significantly easier to use than at the baseline. Further, the perception of mobiles as a useful tool with which to collect data underwent an even greater positive shift. The training also increased positive perceptions of the mobile as a useful way in which to collect data. The positive perceptions were linked to the insight that the mobile phones would allow more accurate data to be collected in a timely manner with less administrative burden than paper forms. After 3 months of use, perceptions of both the usefulness and ease of use of mobile phone–assisted questionnaires decreased, but remained above levels reported pretraining. This decrease in perceived usefulness and ease of use is thought to be related to a more realistic view of the device’s potential being realized through practical experience. It is important to note that real world use did not significantly reduce perceptions of either usefulness or ease of use.

#### Participant Group 2: Focus Group With 12 Mothers Living With HIV

All focus group participants were HIV positive and pregnant. Most (9/12) had learned of their HIV status through the PMTCT program during their first pregnancy. For 4 of the women, this was their first pregnancy and only 1 of the 4 already knew that she was HIV positive. The mean age of the focus group participants was 26 years. Despite an estimated household (not per capita) income, in South African rand (R), of R1600 (equivalent to US $177) per annum for rural communities in this district [26], all 12 woman in the focus group had access to a mobile phone at home, with a full two-thirds (8/12) personally owning a phone. The 4 women who did not own a cell phone had access to a sibling’s phone. When asked how important access to a cell phone was for them, all but 1 stated that it was extremely important.

Reflecting on the experience of being interviewed using a mobile phone, a number of women reported initial feelings of anxiety about the multimedia capabilities of the phone. In particular, women were concerned about being filmed or photographed by the camera, which had a lens situated on the back of the phone. One woman said, “I feel it is ok [to be interviewed on the phone], but it is slightly scary. I feel as though you are recording a video of me. But it’s all right. It was not nice when the interviewer held the phone too close to me because I was worried about the phone camera...whether I was being recorded.”

Two important factors mediated this experience. Firstly, the model of Nokia phone used influenced how comfortable the interviewees felt. The less familiar–looking Nokia E61 business phone raised more concerns from participants than the more familiar candy bar–shaped Nokia 2630. Secondly, the interview style adopted by the interviewer impacted significantly on the experience. Although all interviews were one-on-one, some interviewers took a position directly opposite the woman whereas other interviewers adopted a side-by-side style. For those women who experienced the side-by-side interview, being filmed was reported as being less of a concern. Women who were interviewed by an interviewer sitting directly opposite them felt that side-by-side interviewing would have alleviated much of their anxiety about being photographed. The side-by-side approach had the added benefit of allowing women, who reported being curious about the questionnaire, to see what had been asked and captured on the phone screen. One participant said, “I enjoyed that the interviewer let me see the questions and shared with me what was being recorded.” This information was incorporated into the primary trial by training field workers to offer MLH the opportunity to sit alongside them during the interview. Table 2 presents other significant themes that emerged from the analysis.

#### Participant Group 3: Questionnaire About Mobile Phone-Assisted Personal Interviews

From October 2010 to March 2011, Project Masihambisane conducted 708 interviews with 520 participants (some participants completed interviews at 2 different time points). Of these 520 participants, 512 (98.5%) agreed to take part in the mobile phone questionnaire. The mean age of participants was 27 years with participants speaking primarily isiZulu (93.4%). A small minority had either no schooling (1.6%) or a tertiary qualification (5.8%). Of the remaining 92.6%, 36.4% completed primary education, 39.9% started but did not complete secondary schooling, and 16.3% graduated from secondary school. Approximately the same percentage of women were married (10%) as were living alone (11.9%); the others were either married and living together or apart, or single and in a coresident or non-coresident relationship. There was a 60/40 split between women living in rural vs periurban areas with 35.5% having running water on the premises, 59.2% having flush toilets, and 82.6% having electricity.

Despite 42.4% of the sample being unemployed, 84.0% stated that they owned a mobile phone. Most of these handsets were manufactured by Nokia, running on the Symbian operating system and were able to run Java ME applications (87.4%), a version of the Java programming language designed to run on resource-constrained devices such as mobile phones. From self-reports, the mean length of ownership was 3.3 years with a median amount of R30 being spent on airtime per month. Table 3 compares the demographic profile of those who owned vs those who did not own a mobile phone. Chi-square tests found significant associations between age (χ^2^_2_=6.34, *P*=.04), education (χ^2^_2_=33.52, *P*<.001), socioeconomic status (χ^2^_2_=27.65, *P*<.001), occupation (X^2^_2_=8.16, *P*=.004*)*, and housing characteristics (water: χ^2^_1_=9.79, *P*=.002; electricity: χ^2^_1_=9.6, *P*=.002; flush toilet: χ^2^_1_=12.68, *P*<.001), and ownership of a mobile phone. Examining confidence intervals for each demographic category support these results for all but age. Pregnant women with HIV were less likely to own a phone if they were 20 years old or younger, had a primary school education, were in the lowest socioeconomic status group, and did not have household luxuries, such as running water, electricity, or flushing toilets.

On a 3-point scale, the participants rated the experience of the mobile questionnaire as positive, neutral, or negative. Of the 512 participants, 485 (94.7%) reported a positive experience (Table 4). There were 24 (4.7%) neutral responses from participants and 3 (0.6%) who disliked the mobile questionnaire. With respect to questionnaire mode preference, one-third (29.5%) had no preference, two-thirds preferred the mobile questionnaire (67.8%), and 2.7% preferred pen-and-paper questionnaires.

A 1-way ANOVA revealed a significant increase in positive ratings when comparing the number of interviews completed before taking the questionnaire. That is, people who had completed 4 MPAPI interviews before taking the questionnaire were significantly more likely to rate the questionnaire experience positively than those participants taking an MPAPI questionnaire for the first time (*F*_3,508_=6.795, *P*=.009).

### Feasibility

#### Participant Group 3: Questionnaire About Mobile Phone-Assisted Personal Interviews

Over the course of the primary study (July 2008 to April 2010), 13,653 mobile phone–assisted questionnaires were submitted and 13,650 were received by the data center. This number includes the questionnaires that were used to capture 3 full-length health assessments, the patient-carried maternal health cards, and the daily clinic attendance registers. All clinics had good network coverage and questionnaires were received, on average, 4.2 minutes after being completed. Participants were identified through the capturing of 2 unique numeric identifiers. Of the 3012 full-length questionnaire assessments, 57 (1.9%) were not able to be linked to a participant because of errors in the first 11-digit unique identifier. All 57 cases were resolved by referring to the second 5-digit identifier.

**Figure 8:**
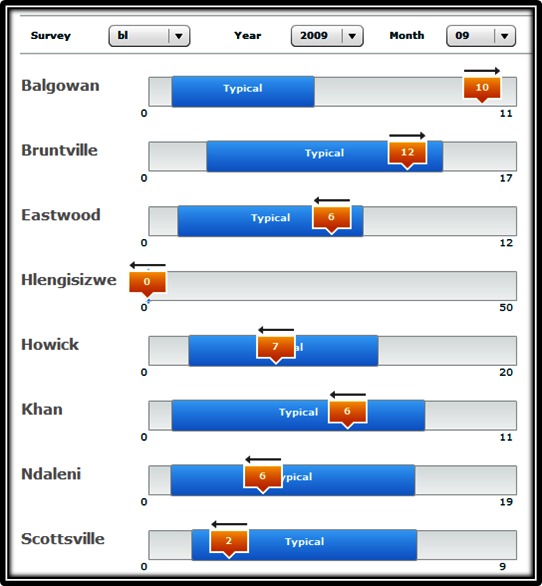
Webpage dashboard example summarizing research assistant interview performance for a baseline interview conducted in September 2009.

Of the 18 mobile phones that were bought for the study, 1 handset was stolen during a mugging and 1 became unusable 13 months into the study after a hardware failure. The remaining handsets are still functional and in use. Over the course of the study, there were very few hardware and/or software challenges. To deal with the possibility that phones would be used for private calls and other electronic communications, mobile phones were loaded with a fixed amount of airtime (US $25) at the beginning of every month. When this allocation was consumed, interviewers were responsible for airtime top-ups. This situation occurred only a few times during the course of the study. During these times, interviewers were able to come to the office and upload the data using a Wi-Fi connection. Automatic processing of submitted data enabled field team leaders to monitor the number of interviews, length of interviews, and quality of interview responses being submitted by interviewers on a Web-based dashboard page. An example from the remote monitoring Web dashboard is presented in Figure 8. At a glance, field coordinators were able to see the minimum and maximum number of interviews per clinic, typical monthly range, current performance, and a trend indicator. These figures could be filtered by questionnaire, year, and month. This quick access to reliable data made it possible to rapidly respond to the ever-changing challenges faced by interviewers working in a rural clinic environment. The approach would also be more easily scaled up to the monitoring of many more clinics than alternative management strategies based on paper and data capturing into a locally stored database.

**Table 2:** Themes emerging from the analysis of the focus group session held with 12 mothers living with HIV (participant group 2).

Theme	Indicative quote^a^
Connectivity	*My phone is important to me as it allows me to communicate immediately with people who want to contact me.* [Gladys Bhengu, 22 years]
	*I use it to get in contact with my family members and people who are living around me, like my neighbors.* [Ethyl Zuma, 36 years]
Safety	*Public phones are far away from where I live. Cell phones [is] convenient and allows one to get help from others through communication.* [Marsha Ntuli, 22 years]
	*...to be able to reach people when I have a problem and vice versa.* [Gladys Bhengu, 22 years]
	*I use it for emergencies...to get help.* [Mavis Sithole, 24 years]
Functionality	*I put reminders in my phone to help remind me of the times when I must take my medication. So I am on time with my medication.* [Ethyl Zuma, 36 years]
Knowledge	*Because you first explain everything before taking any information on the cell phone it will be ok.* [Wendy Zulu, 28 years]
	*As long as the interviewer will explain the study well people won’t mind.* [Ayanda Sithole, 31 years]
Privacy and confidentiality	*They will think that researchers are taking pictures of HIV-positive people to label them.* [Zama Ndwandwe, 33 years]
	*Researchers are using the devices to film people and to put them on television.* [Ethyl Zuma, 36 years]
	*Information will be between the two of us because the phone probably will have a code...I don’t think the data will have a possibility to be lost.* [Wendy Zulu, 28 years]

^a^All names are fictitious.

**Table 3:** Demographic characteristics of sample (N=512).

Respondent characteristics		Do not own phone (n=82)			Own phone (n=430)		
		n	%	95% CI	n	%	95% CI
**Age (years)**							
	≤20	19	23.2	15.4-33.4	55	12.8	10.0-16.3
	21-30	39	47.6	37.1-58.2	247	57.4	52.7-62
	31-40	24	29.3	20.5-39.9	128	29.8	25.6-34.3
**Education**							
	Primary schooling^a^	28	34.1	24.8-44.9	45	10.5	7.9-13.7
	Secondary schooling^a^	53	64.6	53.8-74.1	356	82.8	78.9-86.1
	Tertiary schooling	1	1.2	0.2-6.6	29	6.7	4.7-9.5
**Socioeconomic status**							
	Lower^a^	25	30.5	21.6-41.1	48	11.2	8.5-14.5
	Middle	53	64.6	53.8-74.1	293	68.1	63.6-72.4
	Upper^a^	4	4.9	1.9-11.9	89	20.7	17.1-24.8
**Occupation**							
	Full-time^a^	6	7.3	3.4-15.1	89	20.7	17.1-24.8
	Part-time	27	32.9	23.7-43.7	121	28.1	24.1-32.6
	Unemployed	44	53.7	42.9-64.0	174	40.5	35.9-45.2
	Other	5	6.1	2.6-13.5	46	10.7	8.1-14
**Region**							
	Local government administration	36	43.9	33.7-54.7	171	39.8	35.3-44.5
	Traditional administration	46	56.1	45.3-66.3	259	60.2	55.5-64.7
**Housing**							
	Running water of premises^a^	48	58.5	47.7-68.6	324	75.3	71.1-79.2
	Electricity^a^	58	70.7	60.1-79.5	365	84.9	81.2-88.0
	Flush toilets^a^	34	41.5	31.4-52.3	269	62.6	57.9-67.0

^a^Statistically significant (*P*<.05).

**Table 4:** Questionnaire experience and preference of sample (N=512).

Respondent responses		Do not own phone (n=82)			Own phone (n=430)		
		n	%	95% CI	n	%	95% CI
**Questionnaire experience**							
	Positive	76	92.7	84.9-96.6	409	95.1	92.6-96.8
	Neutral	6	7.3	3.4-15.1	18	4.2	2.7-6.5
	Negative	0	0.0	0-4.5	3	0.7	0.2-2.0
**Questionnaire preference**							
	Phone	50	61.0	50.2-70.8	297	69.1	64.5-73.3
	Paper and pen	5	6.1	2.6-13.5	9	2.1	1.1-3.9
	No preference	27	32.9	23.7-43.7	124	28.8	24.8-33.3
**Questionnaire concerns**							
	Concerned about privacy	9	11.0	5.9-19.6	23	4.1	3.6-7.9
	Concerned about confidentiality	6	7.3	3.4-15.1	21	4.9	3.2-7.4

## Discussion

The present study evaluated the acceptability and feasibility of using mobile phones to collect data from pregnant women living with HIV who were enrolled into the PMTCT program in KwaZulu-Natal, South Africa. Results suggest acceptability of the method by both affected women and the interviewers trained to collect the mobile phone questionnaires. Feasibility of collecting data from remote primary health care facilities was found to be high in this particular context.

### Acceptability

#### Women Living With HIV (Participant Groups 2 and 3)

To prepare for interviewing in a clinic environment pregnant women living with HIV, a focus group with 12 women was undertaken. The experience of completing a face-to-face mobile phone–assisted interview was acceptable to most women. The major concerns raised in the focus group discussion suggested 2 important recommendations. First, if mobile phones are to be used in health settings as data collection tools, it is advisable for health staff to adopt an open side-by-side interviewing style. By placing themselves alongside the women, concerns about data privacy, participant confidentiality, and interview anxiety can be minimized. Secondly, using the informed consent process to explain exactly how data will be collected, how it will be transmitted, and who will have access to this private information may reduce participant anxiety about the MPAPI process. Taking these considerations into the clinic-based questionnaire, 512 women were asked to complete a short questionnaire about their experience. Overall, acceptability of this interview method was confirmed with 94.7% of women, who viewed the experience positively. Only 3 women reported the experience as being negative for them. Given a choice as to whether future interviews should be conducted using mobile phone–assisted interviewing or paper-and-pen, only 2.7% expressed a preference for paper-and-pen.

Ownership of mobile phones among pregnant MLH, although high at 84%, was significantly associated with age, education, socioeconomic status, and occupation. This has both positive and negative implications. Positively, the potential of mHealth to reach MLH was confirmed with 8 out of every 10 women owning a mobile phone. However, caution must be exercised to ensure that mHealth programs, which often aim to increase access to marginalized and underresourced individuals, do not inadvertently further disadvantage the most disadvantaged MLH.

#### Interviewers (Participant Group 1)

Perceived ease of use and perceived usefulness of MPAPI were consistently rated highly by clinic-based interviewers. It is not clear whether this rating is in response to a dislike of paper-based data collection or an affinity for mobile phones. Although perceived ease of use and perceived usefulness dropped after 3 months of field use, they remained above pretraining perceptions. The decrease is likely linked to the fact that real world use is often more limited that portrayed under the ideal conditions made possible in a controlled training environment. Nevertheless, the decrease was not significant and remained high for both perceived usefulness and ease of use. This finding suggests that after extended clinic-based use, the mobile phone–assisted interviews remained useful and easy. Training was found to significantly increase perceived ease of use and perceived usefulness and highlights the importance of providing a comprehensive training package to familiarize interviewers with both the concepts and practice of MPAPI.

### Feasibility

The feasibility of using mobile phone–assisted interviews as part of a clinic-based PMTCT program was monitored for 21 consecutive months. During this time, over 13,000 data forms were submitted with only 3 reported as being sent by interviewers and not received. Very few phones were lost, broken, or stolen and network coverage was good at all 8 clinic facilities. A number of strategies were used to control airtime use, the most effective being a fixed amount of airtime loaded onto the handset at the beginning of the month and interviewers topping up if they depleted this amount. Other strategies included interviewers requesting small amounts of airtime as they needed, weekly checks by a coordinator of what numbers had been dialed, and a usage policy that outlined what the phone may and may not be used for. Initial concerns about charging handset batteries were unfounded as all clinics had electricity.

These limited challenges were supplemented by a number of perceived benefits. The management of geographically dispersed interviewers was improved through the use of real-time data presented in visually appealing summaries. This allowed problems to be flagged much quicker than if data was captured on paper and collected from the clinics on a monthly basis. Although not used in this study, the ability to geotag questionnaire responses could also assist in the management of community health workers providing home-based care. Questionnaire management was also found to be improved with a virtual dashboard giving valuable up-to-date information on key project indicators. Whenever a new questionnaire needed to be introduced or changes made to an existing questionnaire, updates could be rolled out immediately to each clinic without the need to recall out-of-date paper forms. This problem besets current paper-based registers. Whenever a new indicator needs to be collected or a change in indicator definitions occur, old forms continue to be submitted by facilities who either do not receive the updated forms or prevent wastage by continuing to use old form stock. Finally, this study found it feasible to track and link the PMTCT data of participants over a 10- to 14-month period. Although 2% of the records could not be linked because of data entry errors in the unique identifier, the use of a second identifier resolved these cases.

### Relevance to the Prevention of Maternal-to-Child Transmission Cascade

Paper-based records that inform the KwaZulu-Natal District Information System have been shown to be of poor quality and in a format that make the tracking of women through the PMTCT cascade difficult [15]. Proposed as an alternative in other settings [19,27], little is known about the acceptability and feasibility of using mobile phones to collect data from WLH in this setting. The findings presented here suggest that MPAPI is indeed acceptable to both MLH and interviewers. The use of mobile phones for data collection also proved feasible and provided a number of advantages to the data collection process over traditional ways of collecting data in a rural clinic setting. One key benefit for the PMTCT program was the ability to link women’s data over a number of visits. This made it possible to track enrollment, uptake, and loss of individual women through the PMTCT cascade.

### Limitations and Future Work

The results obtained in this study suggest that MPAPI may be both feasible and acceptable to use within the South African PMTCT program. South Africa is unique among African countries in its broad mobile network coverage, well-maintained mobile infrastructure, and electrification of rural health facilities. How well this approach to the collection of PMTCT data would perform in the context of other low- and middle-income countries is, therefore, a limitation of this work.

The bidirectional communication abilities of mobile phones also holds promise as a way to provide regular and relevant feedback to clinics. With a large proportion of women owning mobile phones, future studies should explore the acceptability and feasibility of extending PMTCT data collection into the home using mobile phone–assisted self-interviewing.

## Abbreviations

ANOVA: analysis of variance
API: application programming interface
HIV: human immunodeficiency virus
MLH: mothers living with HIV
MPAPI: mobile phone-assisted personal interview
PMTCT: Prevention of Maternal-to-Child Transmission
